# Resistance to Immune Checkpoint Blockade in Uterine Leiomyosarcoma: What Can We Learn from Other Cancer Types?

**DOI:** 10.3390/cancers13092040

**Published:** 2021-04-23

**Authors:** Wout De Wispelaere, Daniela Annibali, Sandra Tuyaerts, Diether Lambrechts, Frédéric Amant

**Affiliations:** 1 Department of Oncology, KU Leuven (University of Leuven) and Leuven Cancer Institute (LKI), 3000 Leuven, Belgium; wout.dewispelaere@kuleuven.be (W.D.W.); daniela.annibali@kuleuven.be (D.A.); sandra.tuyaerts@uzbrussel.be (S.T.); 2 Division of Oncogenomics, Antoni Van Leeuwenhoek—Netherlands Cancer Institute (AvL-NKI), 1066 CX Amsterdam, The Netherlands; 3 Laboratory of Medical and Molecular Oncology (LMMO), Department of Medical Oncology, Vrije Universiteit Brussel (VUB), Universitair Ziekenhuis Brussel (UZ Brussel), 1090 Brussels, Belgium; 4 Laboratory for Translational Genetics, Department of Human Genetics, KU Leuven (University of Leuven), 3000 Leuven, Belgium; diether.lambrechts@kuleuven.vib.be; 5 VIB Center for Cancer Biology, Flemish Institute for Biotechnology (VIB), 3000 Leuven, Belgium; 6 Centre for Gynecologic Oncology Amsterdam (CGOA), Antoni Van Leeuwenhoek—Netherlands Cancer Institute, University Medical Center (UMC), 1066 CX Amsterdam, The Netherlands; 7 Department of Obstetrics and Gynecology, University Hospitals Leuven (UZ Leuven), 3000 Leuven, Belgium

**Keywords:** uterine leiomyosarcoma, high-risk gynecological tumors, immune checkpoint blockade, resistance, combinatorial treatment strategies

## Abstract

**Simple Summary:**

Immune checkpoint blockade (ICB) has emerged as a very promising therapeutic option for patients, demonstrating unprecedented, durable responses in several difficult-to-treat cancers. Despite research indicating a strong potential for ICB in uterine leiomyosarcomas (uLMSs), a clinical trial assessing response to ICB monotherapy in uLMSs showed no clinical benefit. Resistance to ICB has been studied extensively in a variety of tumor types, but the resistance mechanisms explaining the lack of response to ICB in uLMSs remain largely unexplored. By elucidating and targeting mechanisms of resistance, treatments can be tailored to improve the effectiveness of ICB and accelerate its clinical implementation. Therefore, in this review we will explore what is known about the immunosuppressive microenvironment of uLMSs, link these data to possible resistance mechanisms extrapolated from other cancer types, and discuss potential therapeutic strategies to overcome resistance.

**Abstract:**

The onset of immune checkpoint blockade (ICB) therapy over the last decade has transformed the therapeutic landscape in oncology. ICB has shown unprecedented clinical activity and durable responses in a variety of difficult-to-treat cancers. However, despite these promising long-term responses, a majority of patients fail to respond to single-agent therapy, demonstrating primary or acquired resistance. Uterine leiomyosarcoma (uLMS) is a rare high-risk gynecological cancer with very limited treatment options. Despite research indicating a strong potential for ICB in uLMS, a clinical trial assessing the response to immunotherapy with single-agent nivolumab in advanced-stage uLMS showed no clinical benefit. Many mechanisms of resistance to ICB have been characterized in a variety of tumor types, and many more continue to be uncovered. However, the mechanisms of resistance to ICB in uLMS remain largely unexplored. By elucidating and targeting mechanisms of resistance, treatments can be tailored to improve clinical outcomes. Therefore, in this review we will explore what is known about the immunosuppressive microenvironment of uLMS, link these data to possible resistance mechanisms extrapolated from other cancer types, and discuss potential therapeutic strategies to overcome resistance.

## 1. Introduction

Uterine leiomyosarcoma (uLMS) is the most common subtype of uterine sarcoma (US), with an annual incidence rate of 0.64/100,000 women, accounting for almost 60% of all US cases [1]. Although rare, uLMS entails substantial mortality due to frequent recurrences and distant metastases, with a 5-year overall survival ranging from 57% for stage I to 16% for stage IV [2]. The standard treatment for early-stage uLMS consists of complete hysterectomy [3,4]. In advanced-stage, recurrent, or metastatic disease, chemotherapy remains the mainstay of treatment, but currently no highly effective agents are available [5]. Primary chemotherapeutic options include single-agent or combinatorial doxorubicin-, ifosfamide-, gemcitabine-, and docetaxel-based regimens [3,6]. The overall response rates (ORR) of these chemotherapeutics range between 25% and 38% [5], with a median time to progression of 4.4 to 6.7 months and an overall survival (OS) of less than 2 years [1,7]. These data clearly illustrate the urgent need for new therapeutic options to improve the clinical outcome of advanced-stage, recurrent, and metastatic uLMS patients.

In this regard, the recent success of immune checkpoint blockade (ICB)-based therapies in a variety of difficult-to-treat cancers raises the question of whether such therapies would be applicable in uLMS [8]. Immune checkpoints are inhibitory regulators of the immune system, crucial to maintaining self-tolerance and controlling the extent and duration of immune responses. When engaged by their respective receptors on tumor-infiltrating T cells, they negatively regulate T cell function to dampen the immune response [9]. These immune checkpoints are often overexpressed in the tumor microenvironment (TME) and compromise the immune system’s ability to mount an effective antitumor response. ICB removes these inhibitory signals, enabling the immune system to mount a long-term durable and effective antitumor response [10]. Several biomarkers indicating a potential for ICB have been reported in uLMS. For instance, abundant expression of programmed cell death-ligand 1 (PD-L1), which has been correlated with immune response and is currently used as a biomarker for ICB therapy in other tumor types, has been observed on tumor cells and tumor-associated immune cells in up to 70% of uLMSs [8,11,12,13]. Additionally, a substantial fraction of uLMSs show elevated levels of tumor-infiltrating lymphocytes (TILs), widely recognized as a predictive biomarker for response to ICB [12,13,14]. Finally, uLMSs have a substantial tumor mutational burden, a genomic biomarker that predicts a favorable response to ICB [8,15]. The potential benefit of ICB in uLMS was illustrated by George et al., who reported the case of a treatment-naïve metastatic patient who received pembrolizumab (anti-PD-1) monotherapy and experienced complete disease remission for over 2 years following resection of one treatment-resistant metastatic lesion [16].

Despite these data indicating the putative therapeutic potential of ICB in uLMS, nivolumab (anti-PD-1) monotherapy or in combination with ipilimumab (CTLA-4 inhibitor) showed limited to no therapeutic benefit in clinical trials [1]. These conflicting results illustrate that response to ICB is very complex and heterogeneous. Many mechanisms of resistance to ICB have been characterized in a variety of tumor types, and many more continue to be uncovered. However, resistance mechanisms against ICB in uLMS remain largely unexplored. Therefore, in this review, we will explore the known immunosuppressive microenvironment of uLMS and the role it could play in conferring ICB resistance by extrapolating resistance mechanisms known to operate in other cancer types. Here, we want to stimulate research towards unraveling the mechanisms of resistance to ICB in uLMS and encourage the search for new therapeutic strategies to overcome resistance.

## 2. Possible Mechanisms of Resistance to ICB in uLMS

One of the greatest challenges in the field of cancer immunotherapy is to unravel the complex resistance mechanisms and the development of effective combination treatment modalities able to overcome the resistant phenotype [17]. Resistance can be either primary, in which the patient never responds to therapy, or acquired, if the patient initially responds but relapses after a period eventually [18]. Resistance can also be classified as tumor intrinsic or extrinsic. When the tumor cells alter processes related to gene expression, immune recognition, and cell signaling, these are considered tumor-intrinsic resistance mechanisms. Tumor-extrinsic resistance mechanisms occur external to tumor cells. These include the recruitment and activation of immune-suppressive cells to the TME that interfere with the T cell activation process [19].

### 2.1. Immunosuppressive Tumor Microenvironment

The TME is the environment around a tumor, consisting of blood vessels, various immune and stromal cells, extracellular matrix, and cytokines and plays an important role in response to therapy. Immunosuppressive cells, along with inhibitory cytokines in the TME, can undermine the antitumor immune response [20]. The immune cell infiltrate of uLMS has been shown to include several immunosuppressive cell populations, such as regulatory T cells (Tregs), myeloid-derived suppressor cells (MDSCs), and tumor-associated macrophages (TAMs) [12,14,21]. These immunosuppressive cells physically or functionally interact with immune effectors to cause their inhibition and play a role in conferring resistance to ICB [22].

#### 2.1.1. Regulatory T Cells

The suppressive mechanisms used by Tregs to dampen the antitumor immune response can be grouped into four basic “modes of action”: producing immunosuppressive cytokines, inducing cytolysis in effector T cells (Teffs), metabolic disruption, and modulating dendritic cell (DC) maturation and activation [23]. High numbers of Tregs in the TME is associated with poor prognosis in patients with melanoma, head and neck squamous cell carcinoma, and pancreatic, breast, colorectal, ovarian, and lung cancers [24]. Furthermore, an increased ratio of Tregs to Teffs has been associated with poor response to ICB in pancreas cancer murine models, and the inability to decrease the ratio of Tregs to Teffs may result in resistance to immunotherapy [25,26]. By analyzing the inflammatory infiltrate of 21 uLMS cases, Manzoni et al. found that the lymphocyte component, representing 3–29% of the total inflammatory infiltrate, was composed of 30% ± 22% CD4+ T cells, 62% ± 23% CD8+ T cells, and 9% ± 8% natural killer (NK) cells. CD4+ T cells were 68% ± 36% FoxP3+ and stained largely negative for activation markers (OX40, CD69, and CD32) [14]. Together, these data indicate the presence of a large population of Tregs in the TME of uLMS and suggest they may play a role in the observed resistance to ICB.

#### 2.1.2. Myeloid-Derived Suppressor Cells

MDSCs are another type of regulatory cells capable of promoting immune evasion and tumor growth [8]. The mechanisms whereby these cells mediate their immunosuppressive effects encompass inhibition of Teffs in a contact-dependent manner, via the expression of immunosuppressive receptors on their surface, secretion of anti-inflammatory cytokines, and deprivation of essential metabolic substrates from the TME [27]. Clinical studies have demonstrated that an increased presence of MDSCs in the TME correlates with poor response to ICB [28]. Accordingly, disrupting CXCR2-mediated MDSC trafficking to the TME was shown to enhance anti-PD-1 therapy response in a murine model of rhabdomyosarcoma [29]. A study mapping the immunosuppressive microenvironment in US, including uLMS, showed that these tumors are highly infiltrated by MDSCs. One of the mechanisms by which MDSC exerts its immunosuppressive function is by depleting L-arginine from the TME via the production of arginase-1. However, despite the high degree of MDSC infiltration, US did not show a significant increase in arginase-1 activity compared with normal myometrium [30]. Given the prominent role of MDSC in facilitating immune evasion and resistance to ICB, these findings mandate further investigation into the exact role this immunosuppressive cell population plays in US and uLMS.

#### 2.1.3. Tumor-Associated Macrophages

Macrophages are the most abundant immune cell population in the TME of solid tumors [31]. When recruited to the TME, mature macrophages differentiate into two main groups, namely, classically activated macrophages (M1) and alternatively activated macrophages (M2). Which subtype the macrophages differentiate into is determined by the internal conditions of the tumor, such as the presence of cytokines, chemokines, and other factors secreted by tumor, mesenchymal, and immune cells; the presence of local anoxia; and lactic acid levels in the TME [32]. Particularly, protumorigenic M2-polarized macrophages (TAMs) are known to stimulate tumor cell motility, angiogenesis, and growth and inhibit the antitumor immune response in various ways. The mechanisms used by TAMs to suppress the antitumor immune response include the production of inhibitory cytokines, the expression of inhibitory molecules, apoptotic receptors, and nonclassical human leukocyte antigen (HLA) class I molecules that inhibit the proliferation and cytotoxic activity of NK cells and Teffs [33,34]. The presence of TAMs in the TME has been associated with poor prognosis in a variety of cancers, including lung cancer, gastric cancer, and lymphomas [34]. Several TAM-targeting agents have successfully been tested in the clinical setting, including a selective colony-stimulating factor 1 receptor (CSF1R) inhibitor, which received FDA approval for use in patients with tenosynovial giant cell tumors [35,36].

Several research groups have indicated that uLMSs are associated with high numbers of TAMs. Moreover, evidence indicates that macrophages are actively recruited to the TME due to an increased expression of the macrophage colony-stimulating factor (M-CSF) in uLMS cells [37]. Similar to other tumor types, high numbers of TAMs and increased expression of M-CSF also correlate with tumor progression and are associated with poor prognosis in uLMS [37,38]. Kostine et al. analyzed the immune infiltrate of 87 LMS cases, including 6 uLMSs, and found that 58% and 56% of the tumors were highly infiltrated by CD163+ macrophages and T cells, respectively. Almost all cases expressed HLA-I, while PD-L1 expression was observed in 30% of the cases. The expression of all these immune markers correlated with high tumor grade. Furthermore, when coculturing CD14+ monocytes from healthy donors with LMS cell lines, CD163 was upregulated in the presence of M-CSF-producing LMS cells, suggesting that tumor cells drive macrophage differentiation towards the M2 phenotype [21]. Manzoni et al. analyzed the inflammatory infiltrate of 21 uLMS cases and found that the majority of the infiltrate (64% ± 13%) in all cases consists of myelomonocytic cells, most prominently composed of CD163+ CD68+ CD16+ TAMs (38% ± 13%). In almost all cases, TAMs expressed the activation markers HLA-DR, CD83, and OX40 and the inhibitory ligands PD-L1 and TIM3 [14]. These data indicate that TAMs are very prominent in the TME of uLMS and may play an important role in conferring ICB resistance.

### 2.2. Tumor Immunogenicity

#### 2.2.1. Loss of Neoantigen Expression

The ability of tumors to induce an effective adaptive immune response relies on the immune system being able to distinguish cancer cells from noncancer cells. Neoantigens are immunogenic peptides derived from tumor-specific mutations that can be recognized by the immune system and mount an effective antitumor immune response [39,40]. Response rates of different tumor types to ICB tend to be proportional to their corresponding tumor mutational burden (i.e., their ability to present neoantigens) [8]. The median somatic mutation rate in LMS is 3.09 (range, 1.05–14.76) per megabase, which is comparable to the rates observed in hepatocellular carcinoma, for which the combination of nivolumab and ipilimumab was recently approved as a second-line treatment option [41]. In this context, mechanisms leading to the loss of neoantigen expression by cancer cells may result in resistance to ICB [8].

Recently, George et al. reported the case of a metastatic uLMS patient who experienced complete disease remission on pembrolizumab monotherapy after resection of the primary tumor and one treatment-resistant metastatic lesion [16]. They analyzed the expression profile of the primary tumor, the treatment-responsive metastases, the sole-treatment-resistant metastasis, and germline tissue and identified two neoantigens derived from clonal somatic mutations in MB21D2 and QKI that were expressed at lower levels in the resistant tumor compared with the responding lesions. They demonstrated specific CD8+ reactivity in patient T cells towards these neoantigens in vitro by incubating peripheral blood mononuclear cells obtained from the patient with the neoantigens of interest. These data indicate that resistance to immunotherapies in uLMS can occur via decreased expression of genes that encode immunogenic clonal tumor-specific mutations.

#### 2.2.2. Aberrant Antigen Presentation

The ability of tumor cells to present tumor-associated antigens in the context of HLA class I molecules is required for the generation of an effective anti-tumor-specific cytotoxic T lymphocyte response. Many tumor cells downregulate or completely abolish antigen presentation, thereby evading cytotoxic T lymphocyte-mediated immune surveillance and elimination. Interferon (IFN)-γ-inducible genes (e.g., the proteasome subunits); low-molecular-weight proteins (LMP)2, LMP7, and LMP10; and antigen transporters associated with antigen processing (TAP)1 and TAP2 are required for the T cell-mediated recognition of tumors [42,43]. Immunohistochemistry experiments performed by Hayashi et al. revealed a loss of LMP2 expression in 49/58 uLMS cases [44]. Furthermore, female LMP2 knockout mice spontaneously develop uLMS with a disease prevalence of 37% at 12 months of age, indicating a tissue-specific role of LMP2 in protection from spontaneous neoplasms of the uterus [45,46]. In uLMS cell lines, the loss of IFN-γ-inducible LMP2 expression was found to be attributable to a mutation in the ATP-binding domain of Janus kinase (JAK)1, leading to inadequate phosphorylation of STAT1. Full phosphorylation of STAT1 is required for complete transcriptional activation of LMP2 [47]. Multiple studies have demonstrated that loss of JAK1/STAT1 signaling results in resistance to PD-1 and CTLA-4 blockade through inability to upregulate HLA-I expression [48,49]. These data suggest that impaired antigen presentation in a majority of uLMSs could be an underlying mechanism of resistance to ICB blockade.

However, high-dimensional analysis of the inflammatory infiltrate of 21 uLMS cases performed by Manzoni et al. paints a more nuanced picture. In 11/21 of the cases, they found CD8+ T cells in the infiltrate displaying a phenotype consistent with antigen exposure (CD69, granzyme B, and granulysin) and exhaustion (PD-1, TIM3, VISTA, and CD39). In 8/21 cases, they also found that a fraction of CD8+ T cells (42% ± 18%) expressed TCF7, a transcription factor linked to a tissue-resident memory phenotype. The expression of TCF7 correlated inversely with PD-1 expression. Most of the tumors displaying the aforementioned phenotype expressed HLA-I. The remaining 10 cases showed low levels of lymphocyte infiltration, with T cells displaying a “passer-by” phenotype. All these tumors stained negative or weak for HLA-I with the exception of two cases [14]. These data indicate that about half of the investigated cases express HLA-I molecules and are recognized by T cells. However, these antigen-experienced T cells display an exhausted phenotype, characterized by an impaired effector function. The TME is a complex immunosuppressive network that plays a crucial role in regulating T cell phenotype and function. Cancer cells and immunosuppressive cells, along with inhibitory cytokines in the TME, can drive T cells to differentiate into an exhausted phenotype [50]. As previously discussed, the TME of uLMS is highly immunosuppressive with high numbers of infiltrating Tregs, MDSCs, and TAMs. Consequently, from these data, we can speculate that Teff cells are able to infiltrate the tumor and recognize tumor cells in half of uLMS cases, but the TME drives these T cells into an anergic state, undermining the antitumor immune response.

### 2.3. Tumor-Intrinsic Signaling Pathways

**Figure 1:**
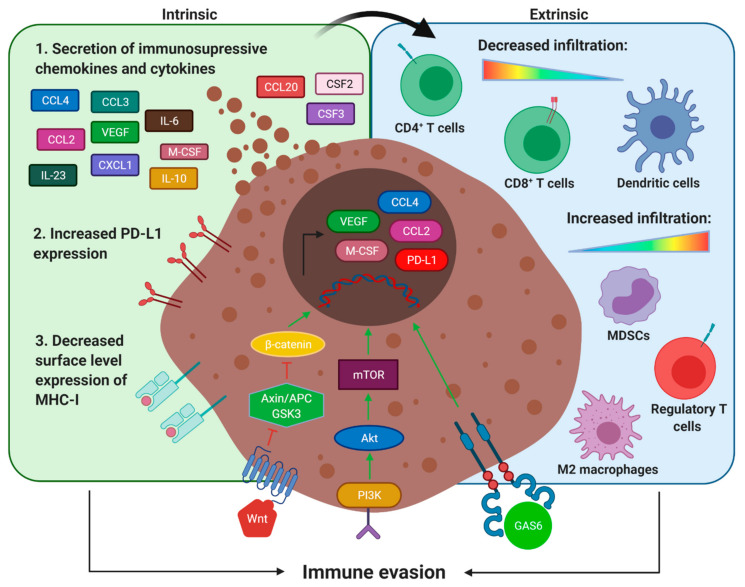
Graphical representation of the different pathways that have a proven role in ICB resistance in other tumor types and could contribute to ICB resistance in uLMS. Overactivation of the PI3K/mTOR, Wnt/β-catenin, and AXL signaling pathways leads to a reduced expression of MHC-I molecules and increased expression of PD-L1 and immunosuppressive cytokines and chemokines. These cytokines and chemokines actively recruit immunosuppressive cell populations to the TME, while hindering the infiltration and activation of Teff cells and DCs. Created with BioRender.com.

Increasing evidence indicates that tumor-intrinsic signaling pathways play a key role in regulating the immune response by altering the tumor cytokine profile and immune cell composition, thereby rendering tumors resistant to ICB [8,51]. A graphical representation of the tumor-intrinsic pathways, enriched in uLMS, which could contribute to inducing tumor-intrinsic or extrinsic resistance to ICB, is shown in Figure 1.

#### 2.3.1. PI3K/mTOR Pathway

The phosphatidylinositol-3-kinase (PI3K)/mammalian target of the rapamycin (mTOR) signaling pathway is a central regulator of cell proliferation, survival, and metabolism [52]. Loss of the tumor suppressor phosphatase and tensin homolog (PTEN), a negative regulator of the PI3K/mTOR pathway, results in constitutive activation of the pathway and tumorigenesis [53]. George et al. reported the case of an uLMS patient with exceptional response to pembrolizumab who experienced complete remission after resection of one treatment-resistant tumor. The resistant tumor harbored a biallelic PTEN mutation and showed increased vascular endothelial growth factor (VEGF) expression, while the responding lesions did not [16]. Strikingly, in melanoma PTEN loss results in constitutive PI3K/mTOR pathway activation, which leads to ICB resistance via induction of VEGF [54]. VEGF suppresses antitumor immunity in a variety of ways, by contributing to the malformation of tumor vessels that can hinder Teff cell trafficking, inhibiting T cell function, interfering with the activation and differentiation of DCs, and actively recruiting Tregs, MDSCs, and TAMs to the TME [55]. In melanoma, VEGF levels were found to be higher in patients not responding to anti-PD-1 therapy, compared with responders, suggesting that it might play a role in resistance [56]. Corroborating this evidence, VEGF inhibition has been correlated with improved response to ICB in melanoma and renal cell carcinoma (RCC) [57]. Besides increased VEGF expression, overactivation of the PI3K/mTOR pathway has also been associated with increased expression of other immunosuppressive cytokines and chemokines, such as CCL20, CXCL1, IL-6, IL-23, and IL-10, and increased expression of PD-L1 on the surface of tumor cells [58,59].

The PI3K/mTOR pathway is overactivated in up to 33% of uLMS cases, predominantly caused by loss of function of negative regulators (PTEN, 17.7%; NF1, 5.6%; TSC1, 3.5%; STK11, 3.5%; TSC2, 3%; PIK3R1, 2.2%) and less frequently through gain of function of positive regulators (RICTOR, 5.9%; IGF1R, 3.0%; AKT2, 1.3%; PIK3CA, 1.3%; AKT1, 0.9%) [2,60,61,62]. Additionally, several studies have shown that VEGF is strongly expressed in uLMS [53,63,64,65]. Together, these findings hint towards a central role of the PI3K/mTOR pathway in conferring resistance to ICB. Therefore, combinatorial treatment strategies of PI3K/mTOR inhibitors and ICB-based immunotherapy could help overcome resistance and greatly enhance the efficacy of immune checkpoint inhibitors.

#### 2.3.2. Wnt/β-Catenin Pathway

Aberrant Wnt/β-catenin signaling is observed in many different tumor types and correlates with increased invasiveness and metastatic potential [66]. Spranger et al. showed that in melanoma, increased levels of β-catenin inversely correlate with the number of TILs, mediated by a decreased expression of the cytokine CCL4 and an inability to recruit CD103+ DCs to the tumor bed, needed for efficient T cell priming. This indicates that in addition to its oncogenic role, constitutive Wnt signaling also plays a role in mediating ICB resistance through tumor immune cell exclusion [67,68]. Kildal et al. found that cytoplasmic and nuclear levels of β-catenin were significantly increased compared with normal myometrium in 36% and 23% of uLMS cases, respectively [69]. The observed correlation between increased β-catenin levels and reduced TIL infiltration in melanoma raises the question of whether similar T cell exclusion mechanisms could be at play in uLMS. Analyzing the inflammatory infiltrate of 21 uLMS cases, Manzoni et al. found that 11 cases showed high levels of TILs, while the remaining 10 cases were classified as an “immune desert” showing very low levels of TILs [14]. Whether the Wnt/β-catenin pathway plays a role in the low levels of TILs observed in a fraction of uLMSs remains to be investigated.

#### 2.3.3. AXL Expression

The TYRO3, AXL, and MER subfamily of receptor tyrosine kinases is involved in carcinogenesis by modulating biology and immune behavior within tumors [70]. These receptors are structurally homologous and share the same ligands: growth arrest-specific 6 (GAS6) and protein S 1 (PROS1). Upon ligation, TYRO3, AXL, and MER initiate downstream signaling, promoting cell survival, proliferation, migration, and adhesion [71]. Immunohistochemical analysis identified TYRO3, AXL, GAS6, and PROS1 to be commonly expressed in uterine and nonuterine LMS, and their expression correlates with a poor outcome [14,71,72,73]. Moreover, TYRO3 and AXL inhibitors reduced cell growth, blocked the cell cycle, and induced apoptosis in LMS cell lines [71]. Besides being critically involved in tumor cell survival, proliferation, metastasis, and invasion, AXL also participates in immunotherapy resistance and immunosuppression regulation. Tumors with high AXL expression can be resistant to immunotherapy through increased GAS6 production, decreased HLA-I expression, and release of myeloid-supporting cytokines (M-CSF, CSF2, and CSF3) and chemokines (CCL3 and CCL4) that promote tumor development and progression by recruiting Tregs, MDSCs, and protumorigenic M2 macrophages to the TME [74,75]. Consequently, pharmacological inhibition of AXL resulted in increased infiltration of total leukocytes, CD4+ T cells, CD8+ T cells, and DCs and decreased infiltration of M2 macrophages in a murine breast cancer model. The recruited T cells had high levels of CD69, Ki67, and IFN-γ, indicating their activation, proliferation, and effector function. The expression of immunosuppressive cytokines, such as arginase-1, TGF-β, and IL-10, was also decreased. These data strongly support that AXL can directly contribute to the immunosuppressive microenvironment in cancer. Moreover, combined AXL inhibition with PD-1 blockade was shown to mount a potent synergistic antitumor response, leading to tumor eradication in breast, colorectal, and ovarian murine tumor models [70]. Thus, AXL-directed therapy in AXL-expressing tumors could hold great potential to subvert innate and/or adaptive resistance and increase the effectiveness of ICB-based immunotherapy. Although the expression of AXL has been demonstrated in uterine and nonuterine LMS, studies specifically assessing its role in the induction of an immunosuppressive microenvironment are lacking.

## 3. Potential Therapeutic Strategies

Recent insights gained in the mechanisms of resistance against ICB have spurred the development of combination strategies using multiple treatment modalities to overcome resistance. The rationale behind these multimodal approaches is based on the possibility to obtain synergistic effects upon targeting several immune escape pathways at once, resulting in improved effectiveness of ICB-based therapies and clinical outcome [76]. Several clinical trials testing a combination of targeted therapy or chemotherapy with ICB in LMS are ongoing. Currently, active clinical trials that also include gynecological LMS in their patient cohorts are listed in Table 1.

### 3.1. Combination with Chemotherapy

Accumulating evidence indicates that anticancer agents—including some conventional chemotherapeutics—can have strong “on-target” (cancer cell-intrinsic) but also “off-target” (cancer cell-extrinsic) immune-potentiating effects, highlighting the rationale for combining these treatment modalities with immunotherapy to achieve superior therapeutic effects [22]. The chemotherapeutics currently used in the clinic to treat advanced-stage uLMS (e.g., doxorubicin, docetaxel, gemcitabine, and ifosfamide) have proven immune-modulatory effects and could therefore be interesting candidates for combination therapies with ICB.

Anthracyclines, such as doxorubicin, can induce immunogenic cell death (ICD). ICD is any type of cell death, which provokes an immunologic response. This relies on the release of damage-associated molecular patterns from dying tumor cells that improves tumor antigen uptake, processing, and presentation by professional antigen presenting cells to T cells. Beyond triggering ICD, anthracyclines reduce the amount of intratumoral and circulating MDSCs and increase the susceptibility of tumor cells to granzyme B [27,77]. Recently, it has been shown that doxorubicin produces strong synergistic antitumor effects in combination with anti-PD-L1 and anti-CTLA-4 mAbs in colorectal carcinoma and fibrosarcoma mouse models, which were mediated through decreased infiltration of Tregs, increased infiltration of CD8+ T cells, and increased expression of costimulatory molecules on DCs [78]. Additionally, doxorubicin was shown to increase the efficacy of adoptive T cell transfer in a murine breast cancer model by selectively eliminating MDSCs in the spleen, blood, and tumor bed [79].

The immune-stimulatory effects of gemcitabine and docetaxel mainly rely on selective reduction of MDSCs in the spleen and tumor bed. In lung cancer-bearing mice, gemcitabine showed cytotoxic specificity for MDSCs, while CD4+ and CD8+ T cells and B cells remained unaffected [80]. Docetaxel administration considerably decreased the MDSC population in the spleen of 4T1-Neu tumor-bearing mice (breast cancer) and promoted the generation of antitumorigenic M1 phenotype macrophages [81,82].

The high degree of MDSC and immunosuppressive TAM infiltration observed in uLMS could be one of the underlying mechanisms to ICB resistance. Therefore, given the immune-modulatory effects of doxorubicin, gemcitabine, and docetaxel, combination therapies of these agents with ICB could be of great clinical benefit in uLMS.

Currently, several clinical trials are testing combinations of chemotherapy and immunotherapy in LMS and uLMS (NCT03899805, NCT03719430, NCT03536780, NCT02406781, NCT04028063, NCT03123276, and NCT03074318).

### 3.2. Combination with Targeted Therapy

Several molecularly targeted therapies are being investigated in combination with ICB, with the aim of blocking oncogenic signaling pathways, decreasing the production of inhibitory cytokines, and decreasing the influx of immunosuppressive cell populations in the TME.

#### 3.2.1. PI3K/mTOR Blockade

PI3K/mTOR pathway activation has been associated with resistance to ICB in several cancer histotypes, including melanoma, prostate cancer, head and neck squamous cell carcinoma, and uLMS. Therefore, targeted inhibition of this pathway has been explored as a strategy to enhance response to ICB [58]. In melanoma mouse models, treatment with a selective PI3Kβ inhibitor improved the efficacy of both anti-CTLA-4 and anti-PD-1 checkpoint blockade and significantly increased the number of infiltrating CD4+ and CD8+ T cells [83]. Similarly, PI3Kγ inhibition was shown to synergize with PD-1 blockade in a mouse model of head and neck squamous cell carcinoma by activating a proinflammatory transcriptional program, promoting the expression of proinflammatory cytokines, and enhancing T cell toxicity [83]. Additionally, genetic and pharmacological inhibition of PI3Kγ has been shown to reduce tumor infiltration of Tregs and MDSCs and reduce tumor growth and metastasis in murine models of melanoma, pancreatic, lung, and breast cancers [58]. Similarly, mTORC1/2 inhibitors have shown to work synergistically with ICB therapy in mouse models of breast cancer and BRAF-mutant melanoma [84,85]. Previously, PTEN deletions and associated constitutive PI3K/mTOR pathway activation have been associated with resistance to ICB in uLMS [16]. Therefore, uLMS with increased levels of PI3K/mTOR activation might also benefit from therapies combining PI3K/mTOR inhibitors and immune checkpoint inhibitors.

Currently, four PI3K inhibitors—copanlisib (pan-class I PI3K inhibitor), duvelisib (PI3Kδ/γ), idelalisib (PI3Kδ), and alpelisib (PI3Kα)—have received FDA approval for a variety of indications, including breast cancer and several lymphoma subtypes [86]. Temsirolimus and everolimus are two mTOR inhibitors that are approved for use in metastatic RCC patients. Interestingly, there is also an ongoing phase I/II clinical trial assessing the efficacy of sapanisertib, an mTORC1/2 inhibitor, as a monotherapy in advanced-stage uLMS (NCT02601209).

#### 3.2.2. Wnt/β-Catenin Inhibition

Wnt/β-catenin signaling mediates resistance to immune checkpoint therapy by blocking cytokines responsible for trafficking immune cells to the TME. Therefore, combining ICB with β-catenin inhibition could be an effective strategy to increase its response to ICB therapy [87]. Ganesh et al. demonstrated that inhibition of CTNNB1, the gene encoding β-catenin, significantly increased T cell infiltration and potentiated the sensitivity to ICB in melanoma, mammary carcinoma, RCC, and neuroblastoma syngeneic mouse models [88]. Kildal et al. discovered that 25% of uLMSs showed increased expression of cytoplasmic and nuclear levels of β-catenin [69], while Manzoni et al. found that almost half of the 21 uLMS samples studied showed low levels of TILs [14]. Whether these high levels of β-catenin correlate with decreased levels of TILs in uLMS remains to be investigated, but these data indicate that Wnt/β-catenin pathway activation could be one of the underlying mechanisms to ICB resistance in a fraction of uLMSs.

There are currently no FDA-approved drugs specifically targeting the Wnt/β-catenin pathway. However, several inhibitors are undergoing clinical trials for a large variety of indications [89]. Unfortunately, these do not include LMS or uLMS.

#### 3.2.3. AXL Inhibition

Combining AXL tyrosine kinase inhibitors with ICB is another strategy that has proven its merits in subverting resistance to ICB. Combined AXL inhibition and PD-1 blockade was shown to mount a potent synergistic antitumor efficacy, leading to tumor eradication in ovarian cancer murine models by inducing the expression of T cell-recruiting chemokines (e.g., CXCL9, CXCL10, and CXCL11), while decreasing the expression of chemokines related to suppressive cell recruitment (e.g., CCL2, CCL3, and CCL4). In concordance with these data, AXL inhibition greatly increased the infiltration, activation, and proliferation of tumor-infiltrating CD4+ and CD8+ T cells and reduced the infiltration of TAMs [70]. AXL expression is commonly observed in uterine and nonuterine LMSs, making it an attractive target for combination therapies with ICB [14,71,72,73].

Several AXL inhibitors have entered clinical trials. However, many target multiple other kinase receptors in addition to AXL. The most advanced AXL-selective inhibitor, bemcentinib, is currently undergoing phase II clinical trials for NSCLC, pancreas cancer, brain and central nervous system tumors, and mesothelioma (NCT03184571, NCT03649321, NCT03965494, and NCT03654833, respectively).

#### 3.2.4. VEGF/VEGFR Inhibition

VEGF inhibitors have also shown great promise in combination with ICB, reversing immunotherapy resistance through normalization of the immune-suppressive TME [90]. Recently, bevacizumab even received FDA approval in combination with atezolizumab and chemotherapy after it showed improved PFS and OS in patients with metastatic NSCLC, and the combination of axitinib, a VEGFR inhibitor, with pembrolizumab was approved as a first-line treatment option for advanced RCC patients [55,91]. VEGF is known to mediate its immune-suppressive effects in part by actively recruiting suppressive cell populations to the TME. Inhibition of VEGF has shown to improve cytotoxic T cell responses and decrease the amounts of tumor-infiltrating Tregs, MDSCs, and TAMs in melanoma and NSCLC murine models.

Several studies show that uLMSs strongly express VEGF and that they are highly infiltrated by Tregs, MDSCs, and TAMs [12,14,21,53,63,64,65]. Therefore, combining ICB with VEGF inhibitors could improve its effectiveness by reducing the amount of tumor-infiltrating suppressive immune cells.

Currently, the effectiveness of sunitinib, a multitargeted tyrosine kinase inhibitor that inhibits VEGFR2 and AXL, in combination with nivolumab is being tested in high-grade dedifferentiated LMS patients, including gynecological LMS (NCT03277924). Sunitinib monotherapy has already received FDA approval for the treatment of gastrointestinal stromal tumors, pancreatic neuroendocrine tumors, and advanced RCC, and is currently undergoing stage I clinical trials for soft-tissue sarcoma (NCT01498835).

### 3.3. Improving the Tumor Microenvironment

Since uLMSs are highly infiltrated by Tregs, MDSCs, and TAMs, disrupting the trafficking, maturation, and function of these suppressive immune cells to the TME may also be beneficial in promoting response to ICB. Zhu et al. demonstrated that tumor infiltration by M2-polarized TAMs and MDSCs could be reduced in a mouse model of pancreatic ductal adenocarcinoma with colony-stimulating factor 1 receptor (CSF1R) inhibitors. Combining CSF1R blockade with anti-PD-1 or anti-CTLA-4 inhibitors showed improved tumor response compared with single-arm anti-PD-1 or anti-CTLA-4 treatment. The study also showed that CSF1R inhibitors can functionally reprogram macrophages to enhance antigen presentation and elicit antitumor T cell responses [92].

Tumors attract immunosuppressive cells to the TME by secreting chemokines that bind to their respective receptors, such as CXCR2 on MDSCs and CXCR4 on Tregs. Disrupting MDSC and Treg trafficking, using inhibitors to CXCR2 and CXCR4, has been shown to enhance anti-PD-1 tumor response in rhabdomyosarcoma and hepatocellular carcinoma murine models, respectively [29,93]. Currently, two phase I clinical trials are ongoing testing combinations of CSF1R targeting agents and immune checkpoint inhibitors in LMS and uLMS patients (NCT04242238 and NCT03277924).

## 4. Conclusions

In this review, we explored the immunosuppressive TME of uLMS and determined which factors could play a role in conferring resistance to ICB based on known resistance mechanisms in other tumor types. uLMSs are characterized by a highly immunosuppressive TME with high numbers of infiltrating immunosuppressive cell populations, such as Tregs, MDSCs, and TAMs. The TME plays a crucial role in regulating T cell phenotype and function. Teffs in the uLMS TME frequently display an exhausted phenotype, which is correlated with an impaired effector function, rendering them ineffective in mounting an antitumor immune response. Evidence suggests that dysregulated tumor-intrinsic pathways, such as the PI3K/mTOR, Wnt/β-catenin, and AXL signaling pathways, might play a key role in ICB resistance by endorsing the expression of an immunosuppressive cytokine profile and facilitating the trafficking of immunosuppressive cell populations to the TME of uLMS. Targeted therapy and chemotherapy have shown to be capable of significantly remodeling the TME, making it more hospitable to T cells. Therefore, combining chemotherapy or targeted treatment with ICB holds great potential to enhance the effectiveness of ICB in uLMS patients.

Due to the intra-/intertumor heterogeneity of uLMS, it is hard to predict the optimal combination therapies for different patients. Currently, biomarkers such as PD-L1, TIL status, and mutational burden are used to predict ICB response. However, these biomarkers are not sufficient to guide combination approaches. Therefore, going forward, biomarker strategies that combine classic approaches with whole-genome sequencing and immune gene signatures to determine which tumor-intrinsic pathways are dysregulated and characterize the immune cell composition of the TME will aid in patient selection and open the door to individualized treatment with ICB as backbone therapy.

## Figures and Tables

**Table 1:** Currently, active clinical trials testing combinations of targeted therapy or chemotherapy with immunotherapy in LMS, including uLMS.

Trial Identifier	Study Phase	Eligible Diseases	Treatment	Primary Outcome	Status
NCT04242238	I	Advanced or metastatic leiomyosarcoma	DCC-3014 (CSF1R inhibitor) + avelumab	MTD * ORR *	Recruiting
NCT03899805	II	Leiomyosarcoma	Eribulin (microtubule-depolymerizing drug) + pembrolizumab	PFS *	Recruiting
NCT03719430	II	Advanced soft-tissue sarcoma for which doxorubicin treatment is considered appropriate	Doxorubicin + APX005M (CD40 agonistic mAb)	ORR *	Recruiting
NCT03536780	II	Metastatic leiomyosarcomas showing progression during or after first-line doxorubicin-based chemotherapy	Gemcitabine + avelumab	ORR *	Recruiting
NCT03277924	I	High-grade (2 or 3) and dedifferentiated leiomyosarcoma	Sunitinib (VEGFR2, PDGFRα/β, KIT FLT3, RET, AXL, CSF1R inhibitor) + nivolumab	PFS *	Recruiting
NCT03241745	II	Preselected MSI */dMMR */hypermutated metastatic or recurrent uterine leiomyosarcomas	Nivolumab	PFS *	Recruiting
NCT02406781	II	Advanced leiomyosarcomas with the presence of tertiary lymphoid structures	Cyclophosphamide + pembrolizumab	ORR *	Recruiting
NCT04028063	II	Advanced and/or metastatic uterine or soft-tissue leiomyosarcomas	Doxorubicin + AGEN1884 (anti-CTLA-4 mAb) + AGEN2034 (anti-PD-1 mAb)	PFS *	Recruiting
NCT04624178	II	Metastatic/unresectable leiomyosarcomas	Rucaparib (PARP inhibitor) + Nivolumab	ORR *	Recruiting
NCT03123276	I	Leiomyosarcoma	Gemcitabine + pembrolizumab	MTD *	Active, not recruiting
NCT03074318	I/II	Metastatic/unresectable leiomyosarcomas	Trabectedin + avelumab	Incidence adverse events	Active, not recruiting

* Maximum tolerated dose (MTD), overall response rate (ORR) defined as the fraction of patients who have complete or partial response to therapy, progression-free survival (PFS), microsatellite instable (MSI), mismatch repair deficient (dMMR).

## Data Availability

No new data were created or analyzed in this study. Data sharing is not applicable to this article.
